# Co-overexpression of the Constitutively Active Form of OsbZIP46 and ABA-Activated Protein Kinase SAPK6 Improves Drought and Temperature Stress Resistance in Rice

**DOI:** 10.3389/fpls.2017.01102

**Published:** 2017-06-26

**Authors:** Yu Chang, Ba Hoanh Nguyen, Yongjun Xie, Benze Xiao, Ning Tang, Wenliu Zhu, Tongmin Mou, Lizhong Xiong

**Affiliations:** ^1^National Key Laboratory of Crop Genetic Improvement, Huazhong Agricultural UniversityWuhan, China; ^2^Institute of Natural Sciences Education, Vinh UniversityVinh, Vietnam

**Keywords:** drought stress, co-overexpression, bZIP transcription factor, SNF-1 related protein kinase, genetic transformation, transcriptome

## Abstract

Drought is one of the major abiotic stresses threatening rice (*Oryza sativa*) production worldwide. Drought resistance is controlled by multiple genes, and therefore, a multi-gene genetic engineering strategy is theoretically useful for improving drought resistance. However, the experimental evidence for such a strategy is still lacking. In this study, a few drought-responsive genes from rice were assembled by a multiple-round site-specific assembly system, and the constructs were introduced into the rice cultivar KY131 via *Agrobacterium*-mediated transformation. The transgenic lines of the multi-gene and corresponding single-gene constructs were pre-evaluated for drought resistance. We found that the co-overexpression of two genes, encoding a constitutively active form of a bZIP transcription factor (*OsbZIP46CA1*) and a protein kinase (*SAPK6*) involved in the abscisic acid signaling pathway, showed significantly enhanced drought resistance compared with the single-gene transgenic lines and the negative transgenic plants. Single-copy lines of this bi-gene combination (named XL22) and the corresponding single-gene lines were further evaluated for drought resistance in the field using agronomical traits. The results showed that XL22 exhibited greater yield, biomass, spikelet number, and grain number under moderate drought stress conditions. The seedling survival rate of XL22 and the single-gene overexpressors after drought stress treatment also supported the drought resistance results. Furthermore, expression profiling by RNA-Seq revealed that many genes involved in the stress response were specifically up-regulated in the drought-treated XL22 lines and some of the stress-related genes activated in CA1-OE and SAPK6-OE were distinct, which could partially explain the different performances of these lines with respect to drought resistance. In addition, the XL22 seedlings showed improved tolerance to heat and cold stresses. Our results demonstrate that the multi-gene assembly in an appropriate combination may be a promising approach in the genetic improvement of drought resistance.

## Introduction

Drought, one of the major abiotic stresses which was faced by the ancestors of modern rice cultivars about ten thousand years ago, is still the major limiting factor of modern rice (*Oryza sativa*) production worldwide, and will still be in the foreseeable future (Sweeney and McCouch, 2007; Lobell and Tebaldi, 2014; Lesk et al., 2016). Despite over 400 million years of evolution of the plant itself, and many generations of crop domestication, it is evident that enormous potential for the genetic improvement of crop yield under unfavorable environmental conditions still remains (Boyer, 1982; Rodriguez and Redman, 2008; Atwell et al., 2014). Since rice is responsible for the feeding of a large part of the world’s population, great efforts have been directed to the genetic and molecular studies and the improvement of its drought resistance in recent decades (Luo, 2010; Hu and Xiong, 2014).

Generally, the generation of a rice cultivar with enhanced abiotic stress resistance is achieved by two approaches: classical genetics and reverse genetics. The former approach, which consists of trait identification, mapping, and elite line construction, is known for being labor intensive and time-consuming (Luo, 2010; Khan et al., 2016). Given the fact that the effects of stress response controlling QTLs are usually minor, very few successful studies using classical genetics approaches have been reported in rice. On the other hand, with the advent of the genomics era, studies on the molecular mechanisms of abiotic stress responses in rice have been substantial, and a large number of candidate genes have been characterized, especially in recent years (Hu and Xiong, 2014; Yoshida et al., 2014; Todaka et al., 2015; Khan et al., 2016).

According to published data, the application of knowledge obtained in reverse genetics studies might be both promising and challenging. It is noteworthy that a large number of reported candidate genes have not been examined for their genetic effects in field conditions or did not exhibit significant effects in their field performance under unfavorable environmental conditions, especially drought stress (Gaudin et al., 2013; Todaka et al., 2015). Except for a handful of successful examples, the remaining exiguous reports stating better field performance with their candidate genes often cannot fully satisfy the demand for agricultural production (Hu et al., 2006; Shen et al., 2015; Todaka et al., 2015; Lee et al., 2016). It is common for these researchers to only test transgenic lines with the overexpression or suppression of a single gene. For example, we have evaluated the drought resistance of several well studied drought-responsive genes in field conditions, and have noticed that the contribution of the overexpression of a single gene was indeed limiting (Xiao et al., 2007, 2009). Thus, just like many researchers have proposed, the strategy of multi-gene assembly, overexpressing two or more genes in a single transgenic plant, might be a practical solution in this situation. Unfortunately, there are very few reports in crop species confirming the application of this strategy, and most of these researches used the marker-assisted strategy and cross breeding instead of genetic transformation (Kang et al., 2016; Shamsudin et al., 2016a,b). The absence of such reports may be partially due to technical challenges in constructing multi-gene assembly vectors, since the construction of multi-gene vectors is often labor intensive and requires specialized vector systems and strains (Li and Elledge, 2005; Chen et al., 2010; Sun et al., 2013). Another challenge for multi-gene assembly is the selection of multiple genes that will have additive genetic effects according to our knowledge.

The abscisic acid (ABA) signaling pathway, which is regarded as the central signaling pathway of abiotic stress response in plants, has been well characterized, and the PYL/RCAR-PP2C-SnRK2 core signaling module has been established (Umezawa et al., 2010; Yoshida et al., 2014). A typical ABA signaling pathway consists of four major components: (i) soluble ABA receptor PYR/PYL/RCAR (Ma et al., 2009; Santiago et al., 2009; Yin et al., 2009); (ii) clade A protein phosphatase 2C, a negative regulator of the downstream signaling pathway by dephosphorylation of SnRK2 protein kinases in the absence of ABA (Umezawa et al., 2009; Bhaskara et al., 2012); (iii) SNF-1 related protein kinase 2 (SnRK2), a positive regulator that can phosphorylate and activate downstream transcription factors (Fujita et al., 2009; Soon et al., 2012); (iv) ABA-responsive element binding factor (ABF/AREB), the third subfamily of bZIP transcription factor that can induce the transcription of ABA-responsive genes (Choi et al., 2000; Yoshida et al., 2010). It has been reported in various plant species that the activity of bZIP transcription factors is regulated by SnRK2s through phosphorylation (Johnson et al., 2002; Kagaya et al., 2002; Kobayashi et al., 2005; Nakashima et al., 2009). Our studies also revealed that the ABA-responsive bZIP transcription factors OsbZIP23 and OsbZIP46 play essential roles in drought resistance in rice, and these transcription factors can be activated by SnRKs including SAPK2 and SAPK6 (Xiang et al., 2008; Tang et al., 2012; Zong et al., 2016). In addition, it has been reported that SAPK6 can also phosphorylate and activate the bZIP transcription factor OREB1, indicating its broad substrate specificity and vital regulatory role in the ABA signaling pathway (Chae et al., 2007). Therefore, it can be predicted that the bZIP transcription factors and SAPKs in the ABA signaling pathway may be potential candidate genes for multi-gene assembly for improving drought resistance.

OsbZIP46, a member of the third subfamily of bZIP transcription factors in rice, has been identified as an abiotic stress responsive gene in the previous study (Tang et al., 2012). Overexpression of the native form of OsbZIP46 did not show significant effect on drought resistance, whereas overexpression of OsbZIP46CA1, a truncated and constitutively active form of OsbZIP46 with the deletion of the negative regulatory domain D, significantly increased the resistance to drought stress both at the seedling and the reproductive stage (Tang et al., 2012). Besides, the genes up-regulated by the overexpression of OsbZIP46CA1 were distinct from those up-regulated by the other members of the third subfamily, implying its unique potential in genetic engineering of drought resistance in rice.

In this study, an overexpression cassette harboring the constitutively active form of *OsbZIP46* (*OsbZIP46CA1*) (Tang et al., 2012) and *SAPK6* were assembled and transformed into the rice cultivar KY131, the main cultivated rice variety in Northeast China, to generate a co-overexpressor (named XL22). The performance of XL22, the corresponding single-gene overexpressors (CA1-OE and SAPK6-OE), and negative transgenic plants (KY131-N) were tested under drought, heat, and cold stress conditions. The results showed that the XL22 transgenic lines exhibited better performance under the drought stress conditions at both the seedling and reproductive stages compared to the CA1-OE, SAPK6-OE, and the negative transgenic lines. The XL22 seedlings were also more tolerant to both heat and cold stresses. Furthermore, transcriptome profiling of XL22, CA1-OE, and SAPK6-OE plants under drought stress treatment revealed that more genes involved in drought response were up regulated in XL22. This study provides supporting evidence of multi-gene assembly for improving drought resistance in rice.

## Materials and Methods

### Generation of Transgenic Rice Plants

**FIGURE 1:**
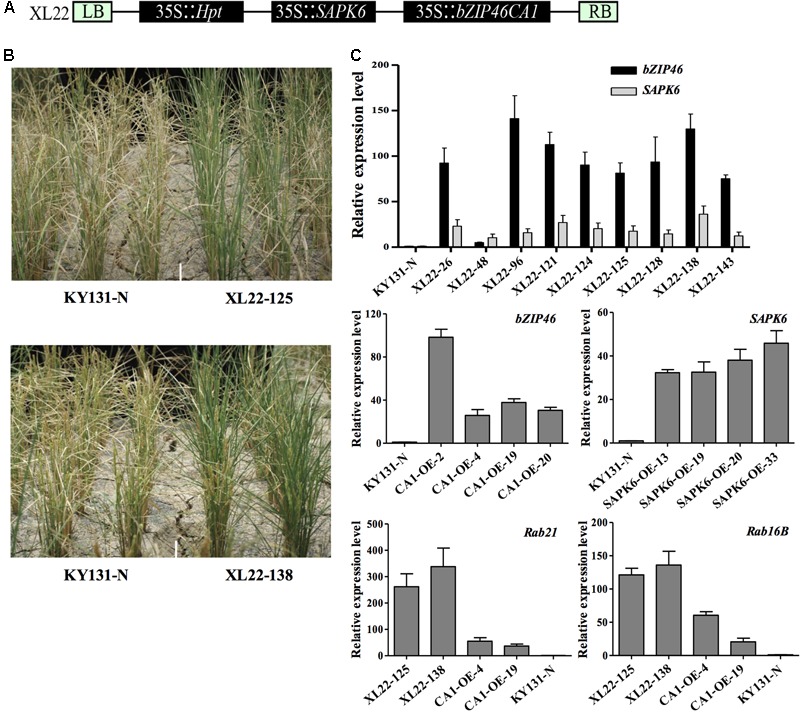
Construction and characterization of transgenic materials. **(A)** Schematic representation of the T-DNA region in XL22. The construct consists of three expression cassettes (35S::*Hpt*, 35S::*OsbZIP46CA1*, and 35S::*SAPK6*), each driven by a CaMV 35S promoter and terminated by a NOS terminator. **(B)** Enhanced drought resistance of the T_1_ XL22 transgenic families during the pre-screening of drought resistance in the field. **(C)** Real-time quantitative PCR analysis of transcript levels in T_2_ generation transgenic seedlings. The relative expression level was calculated via the 2^-ΔΔ^*^C^*^T^ method with *ubiquitin* (LOC_Os03g13170) as an internal control. Error bars indicate the standard deviation (SD) based on three replicates.

To construct XL22, the coding sequences (CDSs) of *OsbZIP46CA1* (Tang et al., 2012) and *SAPK6* (LOC_Os02g34600.1) were amplified from the total cDNA of rice leaves and assembled according to the structure shown in **Figure 1A** via the published procedure of multiple-round *in vivo* site-specific assembly (MISSA) (Chen et al., 2010). The CDSs were also cloned into the binary expression vector pCB2004 (Lei et al., 2007) by the Gateway^TM^ cloning technique^1^ (Invitrogen^TM^) to construct CA1-OE and SAPK6-OE, respectively. The constructs were then introduced into the rice cultivar KY131 (*Oryza sativa L. ssp japonica* cv) via *Agrobacterium*-mediated transformation according to established methods (Hiei et al., 1994; Lin and Zhang, 2005). Negative transgenic plants derived from XL22 transgenic lines (KY131-N) were used as controls.

### RNA Extraction and Gene Expression Analysis

The total RNA of rice leaves was extracted using TRIzol^®^ reagent (Ambion^TM^, Lot No. 15596018) according to the manufacturer’s instructions. The quality and quantity of the total RNAs were evaluated using a NanoDrop^TM^ 2000 (Thermo Scinetific^TM^, Waltham, MA, United States). The DNase treated RNA was reverse transcribed using Moloney murine leukemia virus (M-MLV) reverse transcriptase (Invitrogen^TM^, Lot No. 28025013) and *OsActin* (LOC_Os03g50885) was amplified as a control to identify the quality of the cDNA. The product cDNA was diluted 10 times with ddH_2_O from which 2 μL was used as the template for Real-time quantitative PCR (qPCR) with 5 μL PowerUp^TM^ SYBR^®^ Green Master Mix (Applied Biosystems^TM^, Lot No. A25778), 10 μM forward and reverse primers (0.5 μL for each), and 2 μL ddH_2_O. The qPCR was performed using a Quant Studio 6 Flex Real-Time PCR System (Applied Biosystems^TM^). Thermal cycling conditions for qPCR were as follows: 50°C for 2 min, 95°C for 2 min, followed by 40 cycles of 95°C for 1 s, 60°C for 30 s. The primers used for qPCR are shown in Supplementary Table S1. The relative expression level was calculated via the 2^-ΔΔ^*^C^*^T^ method using *Ubiqutin* (LOC_Os03g13170) as an internal control (Livak and Schmittgen, 2001).

### Southern Blot Hybridization

Genomic DNA was extracted from the leaves of transgenic plants using Plant DNAzol reagent (Invitrogen^TM^, Lot No. 10978021). Southern hybridization was performed according to the standard method (Southern, 2006). In brief, about 4 μg of total DNA was digested overnight with *Eco*R I and then separated by electrophoresis in a 0.8% agarose gel using 1× TAE buffer. After electrophoresis, the gel was denatured and transferred onto a Hybond^TM^-N^+^ membrane (GE Healthcare^TM^, Lot No. RPN1576). The blot was hybridized with the DIG-labeled *Hygromycin phosphotransferase* (*Hpt*) gene probes (for the XL22 lines) and the *phosphinothricin acetyl transferase* (*Bar*) gene probes (for the CA1-OE and SAPK6-OE lines) and exposed to X-ray film for signal detection. DNA probe preparation, hybridization, membrane washing, and signal detection were performed according to the protocols within the PCR DIG Probe Synthesis Kit (Roche^TM^, Lot No. 11636090910). The primers used for probe amplification are listed in Supplementary Table S1.

### ABA Sensitivity Test of the Transgenic Plants

For ABA treatment of the transgenic plants, seedlings from the transgenic lines and KY131-N were shelled and disinfected (through treatment with 75% ethanol for 2 min, 0.15% HgCl_2_ for 10 min, and washing in several changes of sterile water) before germinating on 1/2 strength MS (Murashige-Skoog) medium. After germinating for 4 days in a dark culture condition, the seedlings with identical growth status were selected and transferred to a lucent square box (10 plants per line, three repeats) containing normal 1/2 strength MS medium or 1/2 strength MS medium with 3 μM ABA. After growth for 10 days, the phenotype was recorded and the plant length was measured.

### Stress Tolerance Testing of Transgenic Rice at the Seedling Stage

For drought stress testing at the seedling stage, 1-week-old seedlings of the bi-gene and single-gene overexpression lines (12 plants each, three repeats) were grown in a half-and-half manner in barrels filled with a mixture of soil and sand (1:1). At the 4-leaf stage, watering was stopped and the soil water content was monitored by TRIME-PICO32 (IMKO Micromodultechnik GmbH, Ettlingen, Germany) on the basis of time domain reflectometry (TDR). After the mean TDR value was kept below 25 for 7 days, the plants were recovered by ample watering. Then the survival rates were recorded after recovery for 7 days. For heat and cold stress testing at the seedling stage, 1-week-old seedlings of the transgenic lines and KY131-N were grown in barrels filled with paddy soil (the same as which was utilized in drought stress testing, 12 plants each, three repeats). At the 4-leaf stage, the plants were moved into a growth chamber and treated with temperature stress at 42°C for 12 h (for heat stress), or moved into a cold room at 4°C for 5 days (for cold stress) and then returned to normal conditions for 7 days before the survival rate was recorded.

### Water Loss Rate Measurement

The water loss measurement was conducted as described previously (Fang et al., 2015). Detached leaves from the 4-leaf-old rice plants were cut and exposed to air at room temperature and weighed at the designated times. The water loss rate was expressed as (m_0_–m_n_)/m_0_ × 100% (m_n_: weight of the leaves at the indicated time point; m_0_: weight of the leaves at the start point). Leaves from six plants were collected as one of the three replicates for each tested transgenic line.

### Drought Resistance Testing of Transgenic Rice at the Reproductive Stage

For drought stress testing at the reproductive stage, the plants were grown in a paddy field facilitated with a movable rain-off shelter during the summer of 2016 in Wuhan, Hubei Province, China. The transgenic lines and KY131-N were planted following a randomized block design with four families for each overexpressor. Ten plants for each family were planted in two rows (one plot) with a planting density similar to real agricultural fields. A plot of the co-overexpression family was inserted between two plots of the corresponding single-gene transgenic families, and the KY131-N control was planted after every three of such plots to form a block. Drought stress was applied by stopping watering at the booting stage (about 2 weeks before flowering) in the field, and drought stress was considered to take place when the mean TDR value of a block was less than 25. The drought condition was kept for 14 days before re-watering. The mean TDR value was kept between 40 and 60 in the subsequent 30 days to simulate a moderate drought condition. The same set of materials were planted in the fully irrigated paddy field in the same period as controls for the RNA-Seq analysis. Yield, filling rate, panicle number, grain number, biomass, and spikelet number of the drought treated plants were evaluated as previously described (Xiao et al., 2009).

### RNA-Seq and Analysis

In this study, total RNA for the RNA-Seq analysis was extracted from the second upper leaves of ten plants for each transgenic line both exposed to severe drought stress treatment (mean TDR value <25 for 5 days) and grown in a well irrigated field using TRIzol^®^ reagent (Ambion^TM^, Lot No. 15596018) according to the manufacturer’s protocol. Two independent transgenic lines for each overexpressor were sampled in both condition and were regarded as two biological replicates. RNA-Seq was performed by Novogene Bioinformatics Technology Co. Ltd (Tianjin, China) with Hiseq-PE150 (Illumina, Inc. San Diego, CA United States) and the raw data was analyzed using the software package of Transcriptome Analysis with Reference Genomes in the BMKCloud cloud server^2^. The quantification of the gene expression levels was estimated by fragments per kilobase of transcript per million fragments mapped (FPKM) (Trapnell et al., 2010). A value of |log_2_(foldchange)|≥ 1 (FDR < 0.01) was set as the threshold for differentially expressed genes (DEGs). Gene Ontology (GO) enrichment analysis of the DEGs was implemented by the GOseq R packages based on the Wallenius non-central hyper-geometric distribution (Young et al., 2010). Enrichment of the DEGs in the KEGG pathways was analyzed using KOBAS software (Mao et al., 2005). Rice protein–protein interaction (PPI) data from the STRING database^3^ was used to predict the PPIs of these DEGs (Snel et al., 2000). A combined score >0.5 was set as the threshold for the predicted PPI pairs. Then the PPI networks were visualized in Cytoscape (Shannon et al., 2003).

### Rice Protoplast Transformation and Transcriptional Activity Assay

Rice protoplasts were isolated from KY131 seedlings according to the reported method (Xie and Yang, 2013). To assess the transcriptional activity of OsbZIP46FL (the native full length form of OsbZIP46) and OsbZIP46CA1 in the presence or absence of SAPK6 in rice protoplasts, the coding sequences of the corresponding genes were cloned into the *Bam*H I and *Kpn* I sites of the effector vectors. And the reporter construct harboring the gene for Firefly luciferase (fLuc) driven by the 1.7 kb promoter of the OsbZIP46 targeting gene *RAB21* was generated according to the previous study (Tang et al., 2016). The construct containing the Renilla luciferase (rLuc) gene driven by the *Arabidopsis UBIQUITIN3* promoter (Hao et al., 2011) was used as an internal control. The effector, reporter, and internal control plasmids, in a ratio of 5:5:1, were co-transfected into rice protoplasts. The luciferase activities were measured using the Dual Luciferase Reporter Assay System (Promega, Lot No. E1910) according to the manufacturer’s instructions. The relative luciferase activity was expressed as the ratio of fLuc/rLuc.

### Accession Numbers

Sequence data in this article is accessible in the GenBank or EMBL databases with the following accession numbers: OsbZIP46, LOC_Os06g10880; SAPK6, LOC_Os02g34600; RAB16B, LOC_Os11g26780, RAB21, LOC_Os11g26790. The RNA-Seq data were deposited in the Gene Expression Omnibus under accession number GSE98775.

## Results

### Construction and Validation of the Co-overexpression Transgenic Lines

In order to generate transgenic rice overexpressing multiple abiotic stress responsive genes, multi-gene assembly vectors were constructed using the MISSA system (Chen et al., 2010) and were transformed into the rice cultivar KY131 (*Oryza sativa* L. ssp. *japonica*) by *Agrobacterium*-mediated transformation (Lin and Zhang, 2005). The corresponding single-gene overexpression constructs were built using pCB2004 (Lei et al., 2007) as the backbone and transformed by the same method. For an initial screening of the multi-gene combinations, six drought-responsive rice genes were included for the multi-gene assembly in different combinations.

We identified the transgene copy number of all the T_0_ transgenic plants by southern blot (Supplementary Figure S1) and checked the transcription level of each transgene in the mixed leaf tissues of T_1_ families derived from the single copy T_0_ plants using Real-time quantitative PCR (qPCR). Drought resistance was pre-evaluated in the T_1_ generation in a paddy field facilitated with a movable rain-off shelter to find candidate multi-gene overexpression lines with improved drought resistance compared with the corresponding single gene overexpression lines and negative transgenic plants (KY131-N) for further study.

During the pre-evaluation, the transgenic lines of a bi-gene construct XL22, co-overexpressing the constitutively active form of a stress responsive bZIP transcription factor gene *OsbZIP46* (*OsbZIP46CA1*) and a SnRK2 gene *SAPK6*, exhibited better drought resistance based on visual evaluation of leaf death at the reproductive stage (**Figure 1B**). This co-overexpressor, along with the corresponding single-gene overexpressors, was chosen for further study.

**FIGURE 2:**
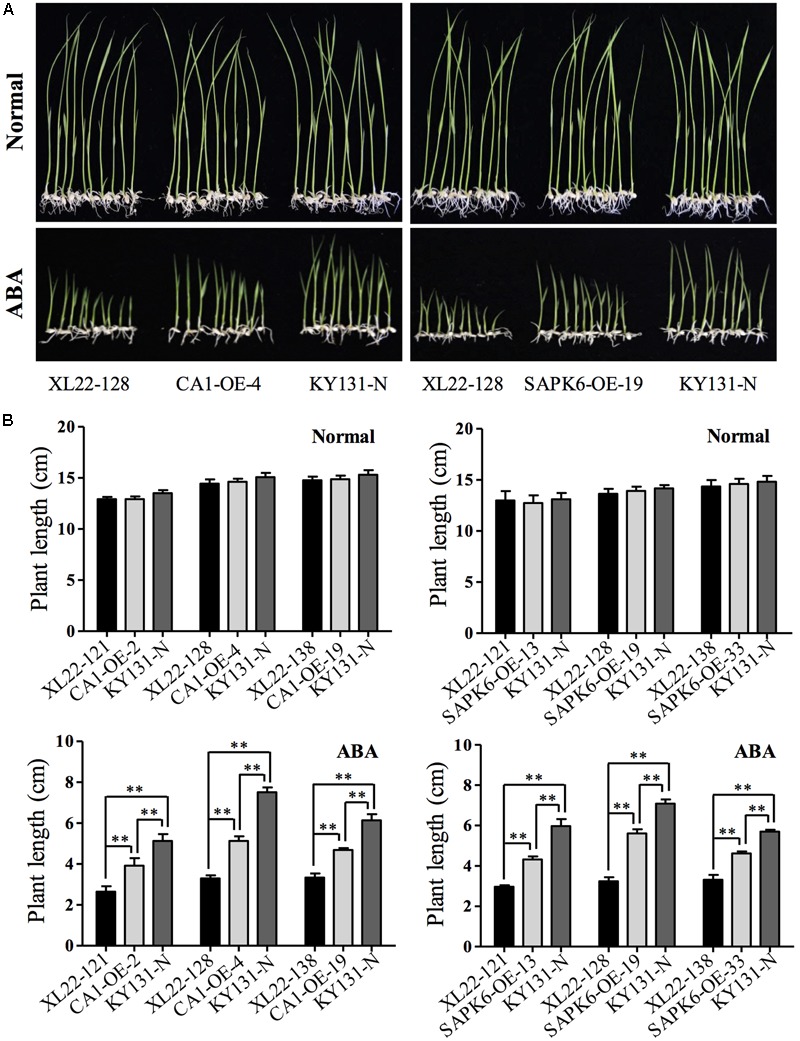
Increased ABA sensitivity of the XL22 seedlings. **(A)** Performance of KY131-N and one of the three tested independent transgenic lines of XL22, CA1-OE, and SAPK6-OE seedlings planted in 1/2 strength MS medium containing 3 μM ABA or normal 1/2 strength MS medium. **(B)** Plant lengths of three independent lines for each construct and KY131-N grown on the normal or ABA-containing 1/2 strength MS medium. The plant length, not including the root, was measured on the 15th day after germination when the photos in **(A)** were taken. Columns represent the average plant length of three independent biological replicates (10 seedlings each) and error bars indicate the standard error (SE). Asterisks indicate the significant difference (^∗∗^*P* < 0.01; ^∗^*P* < 0.05) by Student’s *t*-test).

It has been reported that the CaMV 35S promotor used in this study can cause transgene silencing in some cases (Mishiba et al., 2005; Weinhold et al., 2013). Thus, before starting subsequent abiotic stress testing, we confirmed that *OsbZIP46CA1* and *SAPK6* were overexpressed as expected (**Figure 1C**). Two dehydrin genes, *Rab21* and *Rab16B*, which were up-regulated by the overexpression of *OsbZIP46CA1* in the previous study (Tang et al., 2012) were also strongly induced in the XL22 seedlings (**Figure 1C**). This indicates that at least the cassette of 35S::*OsbZIP46CA1* was functional. Furthermore, we tested the ABA sensitivity of XL22 plants at the post germination stage. As shown in **Figure 2**, after 15 days of growth, the phenotype of all the transgenic lines and KY131-N planted on the control 1/2 strength MS medium was identical, while the CA1-OE lines were hypersensitive to ABA compared with KY131-N, which was consistent with our previous study (Tang et al., 2012). Since SAPK6 is a positive regulator of ABA signaling, the ABA hypersensitive phenotype of SAPK6-OE was also as expected (Chae et al., 2007). The shoot lengths of the XL22 lines were significantly shorter than those of the CA1-OE and SAPK6-OE lines, and KY131-N. This result suggested that all of the constructs were functional as expected for ABA sensitivity, and the co-overexpression of *OsbZIP46CA1* and *SAPK6* may have an additive effect on the ABA response.

### Enhanced Drought Resistance of the XL22 Transgenic Plants

**FIGURE 3:**
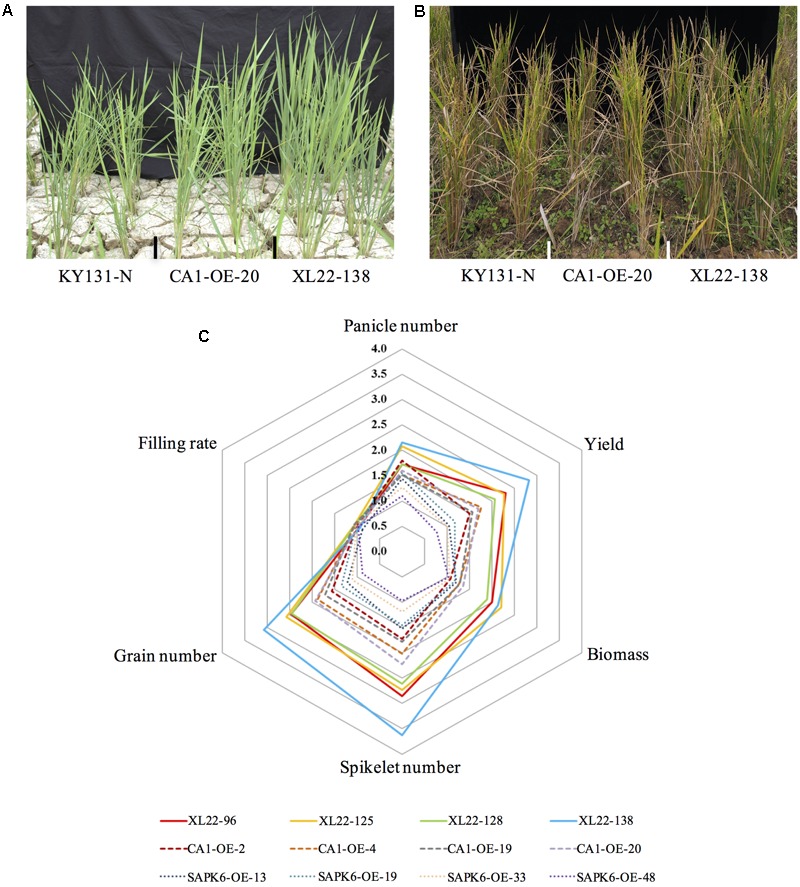
Enhanced drought resistance of the XL22 transgenic lines. **(A)** The plant performance of XL22, CA1-OE, and KY131-N at the beginning of the drought stress treatment. **(B)** The plant performance after one-month of recovery from the drought stress treatment. **(C)** Comparison of the agronomic traits of all the overexpressors measured after the recovery. The radar plot shows the relative average values of panicle number, yield, biomass, spikelet number, grain number, and filling rate, which are normalized to those of KY131-N (set as 1 in the plot).

To evaluate the performance of XL22 under drought stress conditions, four independent transgenic lines for each of the overexpressors (XL22, CA1-OE, and SAPK6-OE) and KY131-N were tested for drought resistance in the same paddy field as in the pre-screening experiment. The water supply was cut off when the plants grew near the reproductive stage and the soil water content was measured on the basis of time domain reflectometry (TDR). Drought stress was considered to take place when the mean TDR value of a block was below 25 (the value was 100 for the water-saturated soil). After 2 weeks of severe drought stress treatment, we re-watered the field by spraying water to maintain the mean TDR value between 40 and 60, which was associated with a moderate drought condition for paddy rice cultivation. After a month of such moderate drought stress treatment, XL22 transgenic lines exhibited better performance in agronomic traits over both single-gene overexpressors and KY131-N (**Figures 3A,B**). As shown in **Figure 3C** and Supplementary Table S2, biomass, grain number, spikelet number, and yield per plant for at least three XL22 lines were significantly higher than those of the single gene overexpressors and KY131-N. The average numbers of panicles for XL22-125 and XL22-138 were also significantly higher than those of the single gene overexpressors and KY131-N. It should be mentioned that some KY131-N plants were completely infertile even after a long period of recovery, while all of the overexpressor plants retained a certain amount of grain yield.

**FIGURE 4:**
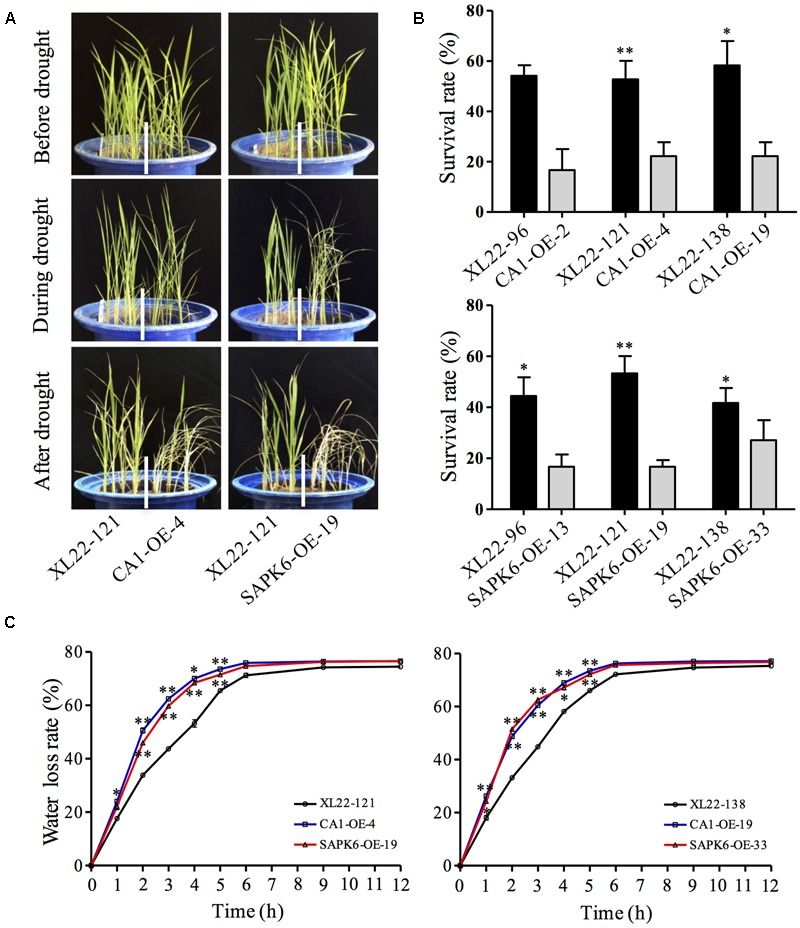
XL22 plants were more resistant to drought treatment at the seedling stage. **(A)** Seedling performance of one of the three tested independent XL22, CA1-OE, and SAPK6-OE lines in pots before, during, and after drought stress treatment. **(B)** Survival rates of drought-treated seedlings after recovery. **(C)** Water loss rates of detached leaves from the seedlings of XL22, CA1-OE, and SAPK6-OE at the indicated time intervals. Error bars in **(B)** and **(C)** indicate the SE based on three independent replicates. Asterisks indicate the significant difference (^∗∗^*P* < 0.01; ^∗^*P* < 0.05) by Student’s *t*-test.

In addition, we tested the drought resistance of 4-leaf-old transgenic seedlings grown in pots. After 7 days of drought stress treatment with the mean TDR value in the pots reached 25, the seedlings were watered for recovery. As is shown in **Figure 4A**, the seedlings of XL22 exhibited weaker symptom of leaf rolling compared to the single gene overexpressors subjected to the same drought stress. And XL22 transgenic lines exhibited significantly greater survival rates when compared with the CA1-OE and SAPK6-OE lines after the recovery (**Figures 4A,B**). Furthermore, the water loss rates of detached leaves from the XL22 lines were significantly lower than those of the CA1-OE and SAPK6-OE lines (**Figure 4C**). These results indicate that co-overexpressing *OsbZIP46CA1* and *SAPK6* can enhance drought resistance better than over-expressing these genes individually.

### The XL22 Transgenic Lines Showed Improved Heat and Cold Tolerance

**FIGURE 5:**
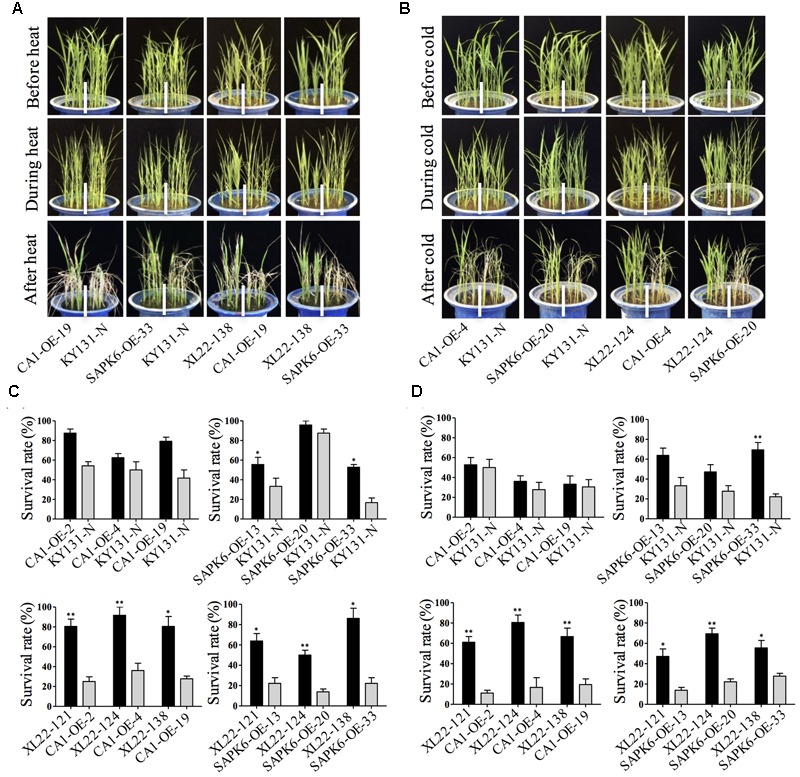
XL22 seedlings were more tolerant to extreme temperature. **(A,B)** The phenotypes of the XL22, CA1-OE, SAPK6-OE lines and KY131-N before, during, and after heat **(A)** and cold **(B)** stress treatment, respectively. **(C,D)** Survival rates were measured after recovery from heat **(C)** and cold **(D)** treatment, respectively. Error bars indicate the SE of three replicates. Asterisks indicate the significant difference (^∗∗^*P* < 0.01; ^∗^*P* < 0.05) by Student’s *t*-test).

In addition to drought stress, extreme temperature stresses including heat and cold are another threat to rice production. To evaluate whether the co-overexpression of *OsbZIP46CA1* and *SAPK6* has an effect on heat or cold tolerance, 4-leaf stage seedlings of three independent lines for each overexpressor and KY131-N were treated with temperature stress at 42°C for 12 h or at 4°C for 5 days, and were recovered for 7 days under normal growth conditions. The results showed that XL22 seedlings were less sensitive to heat or cold stress, and only exhibited slight leaf rolling during treatment (**Figures 5A,B**). In both treatments, the survival rates of the XL22 lines were significantly greater than the single gene overexpressors and KY131-N after recovery (**Figures 5C,D**), indicating enhanced heat and cold tolerance for the XL22 transgenic plants.

### Transcriptome Profiling of the XL22 Transgenic Plants

**FIGURE 6:**
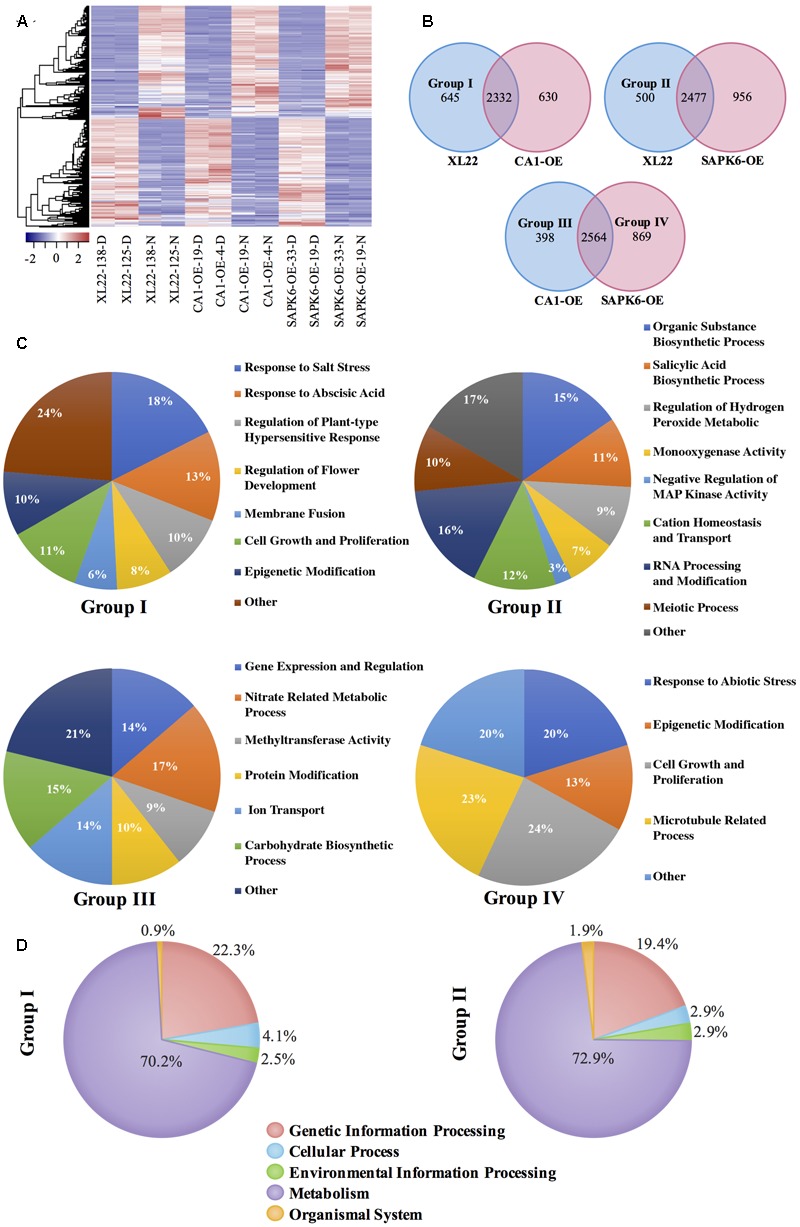
Transcriptome profiling of the transgenic plants. **(A)** Heat map showing the expression patterns of drought-responsive genes in XL22, CA1-OE, and SAPK6-OE plants in normal growth conditions (–N) and drought stress conditions (–D). **(B)** Venn diagrams illustrating the overlap of drought up-regulated genes between XL22 and two single-gene overexpressors. Group I and II represent genes specifically up-regulated in XL22 compared to the up-regulated genes in CA1-OE and SAPK6-OE, respectively. Group III and IV genes were up-regulated in CA1-OE and SAPK6-OE, respectively, when both the single gene overexpressors were compared. **(C)** GO enrichment of the Group I-IV genes. Functionally related GO terms were combined in the pie chart for categorization (details in GO terms were presented in Supplementary Files S4–S7). **(D)** KEGG pathway enrichment of the Group I and II genes.

To elucidate possible molecular mechanisms of the improved drought resistance of XL22, the transcriptomes of XL22, CA1-OE, and SAPK6-OE plants under both severe drought stress and normal growth conditions were analyzed by RNA sequencing (RNA-Seq). The expression profile changes caused by drought stress treatment in the XL22 plants were compared to those in the CA1-OE and SAPK6-OE plants. As shown in **Figure 6A** and Supplementary Files S1–S3, the expression patterns of a large number of genes in the overexpressors were affected by drought stress treatment. With a threshold of |log_2_(foldchange)|≥ 1 (FDR < 0.01), a total of 2977, 2962, and 3433 genes were up-regulated, and 3243, 3514, and 3472 genes were down-regulated in drought-treated XL22, CA1-OE, and SAPK6-OE plants, respectively. More than half of the up-regulated DEGs were common between XL22 and the single-gene overexpressors (2332 for XL22 and CA1-OE, 2477 for XL22 and SAPK6-OE, shown in **Figure 6B**), implicating the conserved functions of these genes in the drought response.

Since OsbZIP46CA1 was previously characterized as a transcriptional activator (Tang et al., 2012), we focused on the up-regulated genes in XL22 for further analysis. There were 645 (Group I) and 500 (Group II) specifically up-regulated DEGs in XL22 compared to CA1-OE and SAPK6-OE, respectively. Gene Ontology (GO) analysis of the Group I and II genes showed that annotated DEGs in several GO terms under ”Biological Process” were significantly enriched (**Figure 6C** and Supplementary Files S4, S5). The top GO terms with the greatest proportions of DEGs in Group I were “Response to Abscisic Acid” and “Regulation of Plant-type Hypersensitive response,” while the top GO terms of Group II genes were “Organic Substance Biosynthetic Process,” “Salicylic Acid Biosynthetic Process,” and “Regulation of Hydrogen Peroxide Metabolic Process”. Furthermore, KEGG pathway enrichment was performed using the Group I and II genes. The results showed that the annotated genes in both groups were mainly enriched in the term of “Metabolism” (**Figure 6D**), suggesting substantial alteration in the metabolisms of XL22 in response to the drought stress treatment.

Drought-inducible genes in CA1-OE and SAPK6-OE were also compared. A total of 2564 genes were up-regulated in both CA1-OE and SAPK6-OE, while 398 (Group III) and 869 (Group IV) genes specifically regulated in CA1-OE and SAPK6-OE, respectively (**Figure 6B**). GO analysis of the Group III and IV genes revealed that the biological and molecular functions of the two groups of genes were largely different. Group III genes were significantly enriched in GO terms ”Gene Expression and Regulation” (21%), ”Nitrate Related Metabolic Process” (17%), and ”Carbohydrate Biosynthetic Process” (15%), while the Group IV genes were mainly categorized into ”Cytokinesis and Cell Proliferation” (24%), ”Microtubule Related Process” (23%), and ”Response to Abiotic Stress” (20%) (**Figure 6C** and Supplementary Files S6, S7). These results suggested that the overexpression of *OsbZIP46CA1* and *SAPK6* might have activated different downstream genes, which may partially explain the different performance of CA1-OE and SAPK6-OE under the drought stress conditions.

**FIGURE 7:**
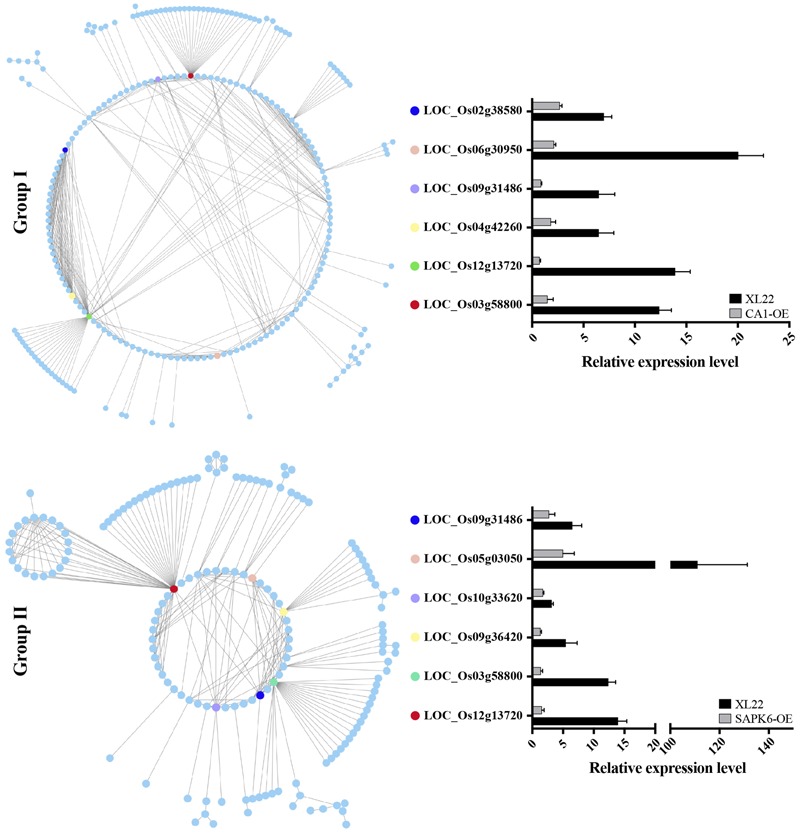
Predicted protein–protein interaction (PPI) network of Group I and II proteins. Six proteins with the greatest number of interactors within each group are highlighted. The histogram shows the relative expression levels of the highlighted genes in response to drought stress detected by qPCR using the same drought-treated samples for RNA-Seq. The relative expression levels were normalized to the corresponding lines grown under normal conditions (arbitrarily set as 1).

To identify the possible relationships of genes within Group I and II, PPI data from the STRING database (Snel et al., 2000) was used to draw PPI networks of Group I and II proteins, respectively. As shown in **Figure 7**, both of the predicted PPI networks were highly complex, with 481 PPIs in Group I and 280 PPIs in Group II. These results indicated closely linked relationships among genes within each group. Of special note, six ”nodes” (proteins labeled in **Figure 7** with MSU locus identifiers) with the greatest number of ”branches” (interaction partners) within each group caught our attention. Three genes (LOC_Os12g13720, LOC_Os03g58800, and LOC_Os09g31486) encoding a PDR ABC transporter, a ATPase, and a DnaK/Hsp70s family protein, respectively, were shared by both group, possibly indicating their vital functions in stress resistance. The remainder of the genes including two protein phosphatase 2C genes (LOC_Os04g42260 and LOC_Os02g38580) in Group I, and Rad51 (LOC_Os05g03050) and Hsp90 (LOC_Os09g36420) family genes in Group II are also closely related to stress according to their predicted functions. Furthermore, quantitative PCR analysis confirmed the up-regulation of these ”node” DEGs in XL22 under the drought stress treatment (**Figure 7**). These results suggested that the up-regulation of genes involved in the PPI networks, especially those node genes, might be involved in the biological processes and/or metabolic networks contributing to the enhanced stress resistance of XL22.

## Discussion

### Co-overexpression of *OsbZIP46CA1* and *SAPK6* Enhanced Drought Tolerance

Since ABA is a critical stress-signaling hormone, some of the ABA signaling components may be promising candidates for engineering drought resistance in crops. A few successful attempts have been made to assess the effects of overexpressing ABA signaling components on drought resistance in rice. For example, overexpression of ABA receptor genes *OsPYL3, OsPYL5*, and *OsPYL9* significantly increased drought resistance in rice at the seedling stage (Kim et al., 2014; Tian et al., 2015). On the other hand, overexpression of *OsPP2C49*, an ABA signaling negative regulator, resulted in a severe dehydration phenotype even under the normal condition (Zong et al., 2016). The positive effects of SnRK2 genes on abiotic stress resistance have been well evaluated in *Arabidopsis thaliana* (Umezawa et al., 2004; Fujita et al., 2009), and overexpression of SAPK4 increased salt resistance in rice (Diedhiou et al., 2008). Furthermore, several members of the third subfamily of bZIP transcription factor have been reported for their positive roles in abiotic stress responses (Fujita et al., 2013). Overexpression of *OsbZIP23* significantly enhanced drought and salt tolerance in rice (Xiang et al., 2008). *OsbZIP46* was characterized as a close homolog of *OsbZIP23* and the overexpression of its constitutively active form OsbZIP46CA1 resulted in improved resistance to drought stress both at seedling stage and reproductive stage (Tang et al., 2012). Therefore, it may be a promising approach to appropriately combine the positive regulators in the ABA signaling pathway to further improve drought resistance. In the study, we simultaneously overexpressed *OsbZIP46CA1* and *SAPK6* in rice cultivar KY131. Our results showed that the co-overexpressor indeed showed better drought resistance than both of the single gene overexpressors at the seedling and the reproductive stages (**Figure 3, 4**).

It is widely accepted that the drought resistance of plants mainly consists of three basic mechanisms: drought tolerance, drought avoidance, and drought escape (Chaves et al., 2002; Chaves and Oliveira, 2004; Yoshimura et al., 2008; Berger et al., 2016). In addition to the three mechanisms, drought recovery also contributes to the final phenotype after exposure to drought stress conditions. In this study, the traits of the tested lines during the process of drought stress treatment were not taken into account because an unexpected high temperature hit the testing field when the majority of the lines were at the flowering stage and simultaneously under severe drought stress treatment (mean TDR value <25), and the filling rate was generally low (0-10% according to observation) among the tested plants. Thus, we extended the recovery period to one month and carefully controlled the soil water content by keeping the mean TDR value of each block between 40 and 60. Although some agronomic traits showed a difference after recovery, the better performance of the co-overexpressor XL22 (as is shown in **Figure 3** and Supplementary Table S2) may not be regarded as the consequence of improved drought recovery. It is known that the cultivation of low land rice varieties including KY131 requires an ample water supply, especially at the reproductive stage (usually with a soil TDR value between 90 and100). However, during the recovery process in this study, the soil TDR value was kept between 40 and 60, which was not a favorable condition for rice growth at the reproductive stage. Thus, the 1 month recovery after severe drought stress treatment was in fact a slight drought condition for the tested lines.

It should be mentioned that the heading date of all the tested transgenic lines and KY131-N were not significantly different under normal and stress conditions, indicating that drought escape via a difference in flowering was not involved in the improved drought resistance of XL22. According to the RNA-Seq data, one of the significantly enriched GO terms in Group II was ”Regulation of Hydrogen Peroxide Metabolic Process” which is a typical physiological process related to drought tolerance (Chaves and Oliveira, 2004; Yoshimura et al., 2008). Besides, out of 9 node genes in the PPI networks, 2 genes (LOC_Os09g36420 and LOC_Os09g31486) encoding heat shock proteins (Hsps) can also be categorized as possible contributors for drought tolerance due to the properties of Hsps in reactivating or sustaining the functions of misfolded proteins in drought stress conditions (Wang et al., 2004; Sarkar et al., 2013; Yu et al., 2015). This indicates that drought tolerance may be one of the major mechanisms for the improved drought resistance of XL22. On the other hand, we noticed that a node gene (LOC_Os12g13720) shared by both Groups encoded a PDR ABC transporter and showed high identity to AtPDR9. It has been reported in *Arabidopsis* that ABC transporters play an essential role in the regulation of stomatal movement and root development, and the mutation of ABC transporter genes resulted in reduced root growth and increased water transpiration and drought susceptibility (Gaedeke et al., 2001; Klein et al., 2003, 2004; Lee et al., 2008; Kuromori et al., 2011). This implies that drought avoidance might also contribute to the improvement of XL22 drought resistance, since drought avoidance is achieved mainly through increasing water uptake and reducing water loss (Berger et al., 2016). Therefore, we propose that the better performance of XL22 in drought resistance may have resulted from the combined effect of enhanced drought tolerance and drought avoidance.

In accordance with the increased drought resistance at reproductive stage, XL22 also showed increased drought resistance at seedling stage when compared with the single gene overexpressors. The leaf rolling of XL22 seedlings was obviously slighter than that of CA1-OE and SAPK6-OE lines (**Figure 4A**), and the water loss in the detached leaves of XL22 was significantly slower (**Figure 4C**). These results imply that the enhanced drought resistance of XL22 may be related to reduced water transpiration. Since OsbZIP46 and SAPK6 are components of ABA signaling, it is reasonable that the co-overexpressor could enhance ABA sensitivity which is generally associated with increased stomatal closure leading to less water loss during the drought stress.

It is noteworthy that, despite superior gains in spikelet and grain number, XL22 was identical to CA1-OE, SAPK6-OE, and KY131-N plants with respect to the grain-filling (or seed-setting) rate (**Figure 3C** and Supplementary Table S2). In our previous study, overexpression of *OsbZIP46CA1* in the rice cultivar Zhonghua 11 (ZH11) also resulted in greater yield without an observed difference in the grain-filling rate after drought stress treatment in PVC tubes (Tang et al., 2012). Thus, we speculate that overexpression of *OsbZIP46CA1* and/or *SAPK6* improves drought resistance mainly through retaining plant growth under water deficient conditions.

SnRK2 family proteins are involved in abiotic responses, and their kinase activities were undetectable but could be activated by osmotic stress (Kobayashi et al., 2004). According to the increased drought resistance of XL22, we suspected that, in addition to the constitutive activation of OsbZIP46CA1, SAPK6 overexpression may further enhance the transcriptional activity of endogenous OsbZIP46 under drought stress conditions. Transcriptional activity assay in rice protoplasts derived from KY131 seedlings showed that the native form of OsbZIP46 was indeed activated to certain extent when co-expressed with SAPK6 (Supplementary Figure S2). Furthermore, according to the presented data (Supplementary Figure S2) and the results of a recent study involving SAPK9 (Tang et al., 2016), OsbZIP46CA1 is a truly activated form of OsbZIP46. However, it cannot be excluded that SAPK6 might activate other members of bZIP transcription factors, as it has been suggested for other SnRK2 proteins (Tang et al., 2012; Wang et al., 2013), which could in turn activate other drought responsive genes. In accordance with this speculation, many genes were specifically up-regulated in SAPK6-OE but not in CA1-OE under the drought stress (**Figure 6B**).

In addition, despite its potential effect on drought stress resistance, the overexpression of SAPK6 alone in KY131 did not result in a greater yield or grain-filling rate according to our data (**Figure 3C** and Supplementary Table S2). A possible explanation may be that the expression level of the native *SAPK6* is relatively high according to public transcriptome profiling data^4,5^, which may partially cover the effect of *SAPK6* overexpression. In addition, there are ten SAPKs in rice, some of which may have functions similar to SAPK6 in the drought response (Kobayashi et al., 2004; Diedhiou et al., 2008). Furthermore, because SAPK6 is a protein kinase, simply overproducing the transcript levels cannot guarantee an increased kinase activity. Future study on the interaction network between SAPK6 and its substrates is required to unveil its precise function.

### Co-overexpression of *OsbZIP46CA1* and *SAPK6* Improves Tolerance to Temperature Stress

It has been reported that pre-treating *Arabidopsis* plants with exogenous ABA could reduce the oxidative damage in recovery from heat stress (Larkindale and Knight, 2002). Overexpression of AREB/ABF transcription factors could increase thermotolerance in *Arabidopsis* (Kim et al., 2004; Zhang et al., 2008; Suzuki et al., 2016). A recent study illustrated that an ABA-inducible heat shock factor HSFA6b, a positive regulator of thermotolerance via activating the transcription of heat stress responsive genes, was transcriptionally regulated by AREB1 in *Arabidopsis* (Huang et al., 2016). Besides, ABA pre-treatment also had positive effect on cold resistance in crop plants such as tomato and wheat (Daie and Campbell, 1981; Lalk and Dörffling, 1985), and the overexpression of ABA receptor-like genes *OsPYL3* and *OsPYL9* could significantly increase cold resistance in rice (Tian et al., 2015). Furthermore, a recent study suggested that the overexpression of OST1, a key SnRK2 protein kinase conducting ABA signaling (Yoshida et al., 2006), enhanced cold tolerance in *Arabidopsis* by activating the CBF-dependent cold signaling pathway (Ding et al., 2015). These findings together indicate that ABA signaling also plays vital roles in the plant responses to temperature stresses.

Despite the identified ABA signaling components contributing to improved heat and/or cold resistance, the effect of *OsbZIP46CA1* and *SAPK6* on heat or cold resistance has not been studied. As is shown in **Figure 5**, the survival rates of most of the CA1-OE and SAPK6-OE lines were only slightly greater (but not statistically significant) than the KY131-N line after heat or cold treatment. This indicates the overexpression of *OsbZIP46CA1* or *SAPK6* alone had very limited effect, if any, on the resistance to abnormal temperature stresses. However, co-overexpression of *OsbZIP46CA1* and *SAPK6* resulted in a significantly elevated survival rate after both treatments (**Figure 5**). Just like in the case of drought stress treatment, it seems that the assembly of *OsbZIP46CA1* and *SAPK6* also had an additive effect on the resistance to unfavorable temperatures. However, it has to be mentioned that the co-overexpression did not seem to contribute to heat resistance at the reproductive stage, since the grain-filling rate of XL22 and the other tested transgenic lines and control plants showed no significant difference in the high temperature condition during the summer of 2016. This result also implies that the effect of the co-overexpression of a given multi-gene combination largely depends on not only the stress type but also the growth stage. It should be also noted that, although the assembly of *OsbZIP46CA1* and *SAPK6* resulted in improved drought resistance, it does not necessarily mean that the assembly of any known stress-related gene has an additive genetic effect on stress resistance. In fact, we have tested several multi-gene assemblies harboring 2–4 different genes with known function in drought resistance, and most of the assemblies showed no significantly improved resistance or even an increased sensitivity to drought stress treatment than overexpressors of a single gene. It is likely that some transgenes may cause antagonistic effects in certain biological pathways when over-expressed together. Additionally, it is common that overexpression of some stress-responsive genes may cause growth repression and/or yield loss. Therefore, it is recommended to gain insights into the molecular mechanism of each candidate gene and optimize the combination before starting a multi-gene assembly project. Meanwhile, more combinations of stress-related genes with known functions should be tested or optimized for a specific abiotic stress at a specific growth stage when facing complex or variable environmental conditions.

## Conclusion

The results in this study demonstrate that co-overexpression of *SAPK6* and the constitutively active form of *OsbZIP46* in the rice cultivar KY131 can further enhance the resistance to drought and temperature stresses compared to the transgenic lines overexpressing the single gene. This multi-gene assembly may be further optimized, for example, using strong stress-inducible promoters, for application in stress-resistance breeding in rice.

## Author Contributions

YC generated the transgenic materials, designed the experiments, and wrote the manuscript, BN performed the experiments, YX analyzed RNA-Seq data, BX and NT provided assistance on vector construction, WZ provided assistance on the drought pre-screening experiment, TM provided vital advice on the article, LX designed the experiments and wrote the manuscript.

## Conflict of Interest Statement

The authors declare that the research was conducted in the absence of any commercial or financial relationships that could be construed as a potential conflict of interest.

## Footnotes

1 https://www.thermofisher.com

2 http://www.biocloud.net

3 http://www.string-db.org

4 http://bar.utoronto.ca

5 http://ricexpro.dna.affrc.go.jp
