# Supplementary material for: Co-overexpression of the Constitutively Active Form of OsbZIP46 and ABA-Activated Protein Kinase SAPK6 Improves Drought and Temperature Stress Resistance in Rice
Source: Front Plant Sci. 2017 Jun 26;8:1102. doi: 10.3389/fpls.2017.01102 (PMC5483469; doi:10.3389/fpls.2017.01102)
Supplement: Supplementary file 1 [file Data_Sheet_1.PDF]

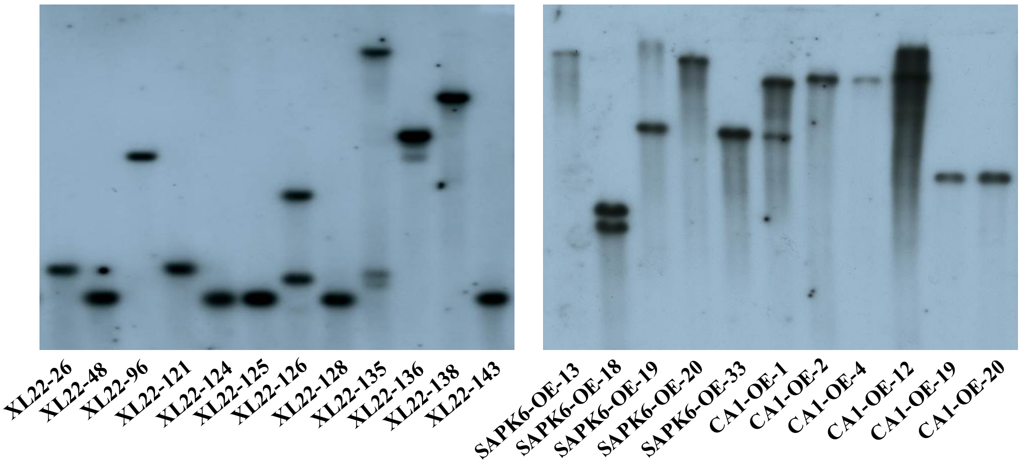

Supplementary Figure 1. Transgene copy number of XL22, CA1-OE, and SAPK6-OE lines determined by Southern blot.

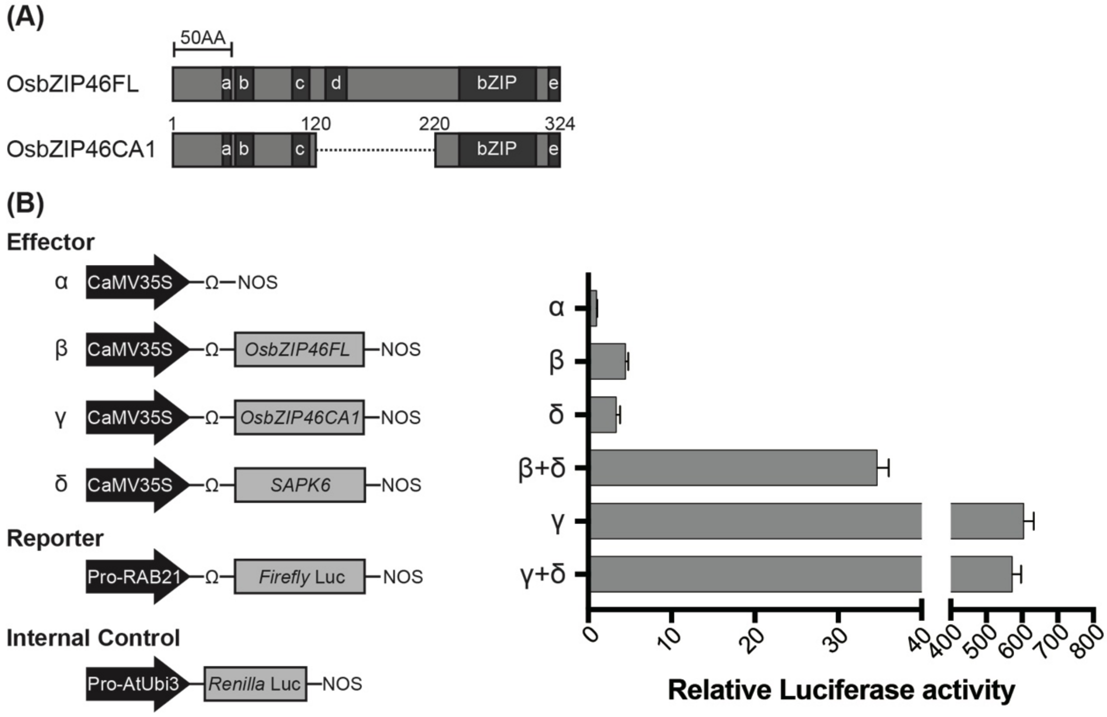

Supplementary Figure 2. Analysis of the transcriptional activity of OsbZIP46. **(A)** Schematic representations of the native full length OsbZIP46 (OsbZIP46FL) and OsbZIP46CA1. Regions of the conserved domains (a-b and bZIP) are highlighted in dark grey. The scale bar indicates the length that represents for 50 amino acid residuals. **(B)** Relative luciferase activities of the two different forms of OsbZIP46, combined with or without SAPK6, in rice protoplasts. Schemes of the constructs used in the transformation are shown on the left. The reporter construct contained the promoter of *RAB21*, which has been identified as a target gene of OsbZIP46, the reporter gene for firefly luciferase (*fLuc*) and a nopaline synthase terminator (NOS). The coding sequences of the tested genes were driven by the CaMV 35S promoter in the effector constructs; and a tobacco mosaic virus enhancer sequence (Ω) was inserted between the CaMV 35S promoter and the translation initiation site. The construct harboring the coding sequence for Renilla luciferase (*rLuc*), which was driven by the promoter of *Arabidopsis ubiqutin3* (Pro-AtUbi3), was co-transfected as an internal control. The relative luciferase activity was calculated based on the fLuc/rLuc ratio; and the value obtained from the transfection of the blank effector (α) was set as 1. Error bars indicate the SD based on three replicates.

Supplementary Table 1. Primers used in this study

Target Name	Primer Sequence		Target Locus
	Sense (5’→3’)	Anti-sense (5’→3’)	
**Primers for qPCR**			
ubiqutin	AACCAGCTGAGGCCCAAGA	ACGATTGATTTAACCAGTCCATGA	LOC_Os03g13170
OsbZIP46	AGCAGGTGGAAATGATACAG	GGTCCAAGTTGCTGAGTGATTC	LOC_Os06g10880
SAPK6	CAAAGACACCACCTCCAT	GTACTCGTCCTCGCTATC	LOC_Os02g34600
Rab16B	AGCTCCAGCTCGTCGTCTGA	GCCAGTGTTCCCCATCATCT	LOC_Os11g26780
Rab21	CGAGCGCAATAAAAGGAAAAA	AGACACGGTCCGTACTGGAGAA	LOC_Os11g26790
Os12g13720	TGGAGCAGTTCAAAGCTTGC	ATGTGCCTGATGTTGCCTTG	LOC_Os12g13720
Os03g58800	CGGGAGTTACATGTGTTCGTTG	TGCCGTTCTTGAACATGTCG	LOC_Os03g58800
Os09g36420	ACCCTGCAAAACTTCTCTGC	AACCAAACTCAGCACAACCG	LOC_Os09g36420
Os10g33620	TTGCAGAGAAGTTGCGGATC	TTTCACCACCGCTGAATTCC	LOC_Os10g33620
Os05g03050	TGAGAAAAGCGCAACAGGTC	TTGGCTGCGTACTTGGTTTG	LOC_Os05g03050
Os09g31486	TTGTCAAGGTGTCAGCCAAG	TTCAGACAAACCGCCTGAAG	LOC_Os09g31486
Os04g42260	AATGCTGGTGATGCTCGTTG	AAAGATACGGAACCTCCTGCAG	LOC_Os04g42260
Os06g30950	ACCTTTCACTGTGCTGTTGG	AACTGAAGCAATGCCCACTC	LOC_Os06g30950
Os02g38580	AGGTTGTTGAGTTCGGCTTG	ACTGGTGCTGGCAATGTTAG	LOC_Os02g38580
**Primers for Probe Amplification**			
Hpt	CTTCTGCGGGCGATTTGT	CGTTATGTTTATCGGCACTTT	-
Bar	TGGTGTAAACAAATTGACGCTT	ACCACTACATCGAGACAAGC	-

Supplementary Table 2. Data on the agronomic traits of all the overexpressors and KY131-N measured after the moderate drought stress treatment in the field.

Transgenic line	Panicle number	Yield (g)	Biomass (g)	Spikelet number	Grain number	Filling rate
XL22-96	8.50 ± 1.43 b	**3.43 ± 0.60 b**	**16.41 ± 4.38 ab**	**281.30 ± 25.37 b**	**174.90 ± 17.65 b**	0.62 ± 0.04 a
XL22-125	**10.30 ± 2.06 a**	**3.38 ± 0.72 b**	**18.14 ± 6.35 a**	**269.90 ± 55.21 b**	**182.10 ± 23.05 b**	0.69 ± 0.08 a
XL22-128	8.50 ± 1.78 b	3.08 ± 0.78 bc	**15.50 ± 2.59 b**	257.40 ± 68.71 bc	**173.70 ± 46.29 b**	0.68 ± 0.11 a
XL22-138	**10.67 ± 2.29 a**	**4.20 ± 1.31 a**	**17.48 ± 3.06 ab**	**358.00 ± 70.30 a**	**217.89 ± 60.58 a**	0.60 ± 0.12 a
CA1-OE-2	8.90 ± 2.38 ab	2.23 ± 0.73 cd	8.78 ± 1.83 cd	169.70 ± 51.82 d	111.10 ± 39.30 cd	0.65 ± 0.08 a
CA1-OE-4	7.50 ± 1.35 bc	2.61 ± 0.38 c	10.39 ± 0.93 cd	198.70 ± 20.58 cd	133.40 ± 16.57 c	0.67 ± 0.07 a
CA1-OE-19	7.50 ± 2.17 bc	2.33 ± 0.60 cd	10.55 ± 2.72 cd	176.10 ± 45.25 d	121.70 ± 34.96 cd	0.69 ± 0.09 a
CA1-OE-20	7.90 ± 1.29 bc	2.52 ± 0.35 c	11.13 ± 1.40 c	219.00 ± 44.75 c	137.20 ± 18.50 c	0.64 ± 0.09 a
SAPK6-OE-13	7.10 ± 1.10 bc	1.54 ± 0.33 d	9.87 ± 1.42 cd	150.70 ± 33.88 de	86.20 ± 20.63 de	0.57 ± 0.07 a
SAPK6-OE-19	7.70 ± 1.95 bc	1.75 ± 0.56 d	9.63 ± 1.56 cd	143.00 ± 35.62 de	95.80 ± 29.62 d	0.66 ± 0.10 a
SAPK6-OE-33	6.30 ± 0.82 c	1.49 ± 0.41 d	8.74 ± 1.83 cd	117.50 ± 17.81 e	78.20 ± 16.92 de	0.66 ± 0.08 a
SAPK6-OE-48	5.44 ± 1.94 c	1.14 ± 0.70 d	8.43 ± 1.94 d	95.44 ± 39.57 e	62.11 ± 34.45 e	0.63 ± 0.15 a
KY131-N	4.95 ± 2.44 c	1.49 ± 0.73 d	8.22 ± 2.95 d	98.63 ± 46.69 e	70.60 ± 35.14 e	0.65 ± 0.25 a

The data is presented as mean ± SD (n=9 or 10 for each overexpressing line and n=40 for KY131-N). Lowercase letters beside each value indicate significant difference calculated via Least Significant Difference (LSD) method at α=0.05. Significantly higher values in XL22 lines are highlighted in bold.
